# The neurological outcome of radiotherapy versus surgery in patients with metastatic spinal cord compression presenting with myelopathy

**DOI:** 10.1007/s00402-017-2817-5

**Published:** 2017-10-14

**Authors:** Keiichiro Iida, Yoshihiro Matsumoto, Nokitaka Setsu, Katsumi Harimaya, Kenichi Kawaguchi, Mitsumasa Hayashida, Seiji Okada, Yasuharu Nakashima

**Affiliations:** Department of Orthopaedic Surgery, Faculty of Medical Sciences, Kyushu University, 3-1-1 Maidashi, Higashi-ku, Fukuoka, 812-8582 Japan

**Keywords:** Metastatic spinal cord compression, Neurological outcome, Myelopathy, Radiotherapy, Surgery

## Abstract

**Purpose:**

While radiotherapy is generally an acceptable treatment for metastatic spinal cord compression, surgical intervention is controversial due to the invasiveness and diversity of diseases in the patients being considered. The ideal treatment, therefore, depends on the situation, and the most acute treatment possible is necessary in patients presenting with myelopathy. We compared the neurological outcomes between radiotherapy and surgery in patients with metastatic spinal cord compression presenting with myelopathy.

**Methods:**

A total 54 patients with metastatic spinal cord compression presenting with myelopathy treated in our institution between 2006 and 2016 were analyzed retrospectively. Twenty patients were selected by radiotherapy alone (radiation group), and 36 patients were selected by decompression and stabilization surgery with or without radiotherapy (surgery group). The neurological outcomes and complications were compared between the two treatment groups.

**Results:**

Seven patients initially in the radiation group underwent surgery because of a substantial decline in their motor strength during radiotherapy. One of the remaining 13 patients (8%) in the radiation group and 30 of the 34 patients (88%) in the surgery group showed improvement in their neurological symptoms (*P* < 0.01). One patient (8%) in the radiation group and 21 patients (62%) in the surgery group were ambulatory after treatment (*P* < 0.01). There were no major complications related to radiotherapy, but surgery-related complications occurred in 9 of 34 (26%) patients, and 6 (18%) patients needed reoperation.

**Conclusions:**

Surgical decompression and stabilization may be required to improve the neurological function in patients with metastatic spinal cord compression presenting with myelopathy. However, the high rate of complications associated with surgery should be taken into consideration.

## Introduction

Metastatic spinal cord compression (MSCC) has been reported to occur in 5–14% of cancer patients [1, 2]. However, the optimum treatment for MSCC remains controversial. Some reports have shown that surgery was superior to radiotherapy alone [2–4], but others describe no significant differences in the outcomes between surgery and radiotherapy [5]. Further complicating matters is the fact that the differences in these results are largely due to differences in the inclusion criteria of registered patients, which hampers the review of the findings.

The radiographic definition of MSCC is compression of the dural sac and its contents, and the compression level (spinal cord or cauda equina) does not matter [6]. The clinical symptoms of MSCC include any or all of the following: back pain, motor weakness, sensory changes, and bladder dysfunction [6]. The term “MSCC” includes several conditions, and many reports have failed to standardize the major neurologic factors to compare the outcome between radiotherapy and surgery. Patients presenting with radiculopathy can be first treated by radiotherapy because there is no urgent need to decompress the spinal cord. However, patients presenting with myelopathy need acute treatment for neurological recovery, as paralysis can proceed rapidly otherwise. The symptom and the compression level of the spinal cord are important factors to consider when deciding on surgical treatment. We, therefore, suggest that the symptoms (myelopathy or radiculopathy) and the compression level of the spinal cord (cervethoratic or lumbar) should be clearly noted when discussing treatment concerning the neurological outcome of patients with MSCC.

We herein report the neurological outcomes following treatment by radiotherapy alone or surgery in patients who presented with myelopathy with spinal cord compression in the cervethoratic region.

## Materials and methods

A total 54 patients with MSCC in the cervicothoracic spine presenting with myelopathy treated in our institution between 2006 and 2016 were analyzed retrospectively. Neurological examinations and magnetic resonance imaging (MRI) were performed at the time of the diagnosis for each patient. Those with certain radiosensitive tumors (lymphomas, leukaemia, and multiple myeloma), metastatic tumors in the lumbar spine, and only pain symptom were excluded. Twenty patients were selected by radiotherapy alone (radiation group), and 34 patients were selected by surgery with or without radiotherapy (surgery group).

The Tokuhashi score [7], Spine Instability Neoplastic Score (SINS) [8], and Frankel grade [9] before treatment were investigated. The neurological recovery rate, complications, and survival after treatment were compared between the two groups. Neurological recovery was evaluated at 1 month after treatment, or at the time of discharge from our institution when the patients were discharged after less than 1 month. Survival was calculated from the first day of the treatment. The neurological status was assessed using the modified Frankel grade [9–11] (Table 1), and an improvement or deterioration of the motor function was defined as an increase or decrease, respectively, in the modified Frankel grade. Ambulatory status was defined when a patient was able to take at least two steps with each foot unassisted, even if a cane or walker was needed [2].

**Table 1:** The modified Frankel grading system

Grade	Neurological status
A	Complete: no motor or sensory function
B	Sensory only: some sensation preserved, no motor function
B1	Touch sensation remains in only sacral lesion
B2	Touch sensation remains in lower extremity
B3	Pain sensation remains in sacral lesion or lower extremity
C	Motor useless: some sensory and motor function, but motor function not useful
C1	Unable to flex the hip and knee from supine (hip flexors 0–2)
C2	Able to flex the hip and knee from supine (hip flexors 3–5)
D	Motor useful: sensory function preserved, motor function weak but useful
D0	MMTs of lower extremity are 4–5, but because of an acute phase, it is impossible to test the walking ability
D1	Able to walk with a walker, but not practiced, usually use a wheel chair
D2	Independent gait with a cane
D3	Independent gait without a cane
E	Normal: normal sensory and motor function (hyperreflexia and numbness are permitted)

Radiotherapy for both treatment regimens was delivered in a split course (3 Gy × 10), and the patients treated with surgery plus radiotherapy were all treated with surgery followed by radiotherapy. The surgery was laminectomy of the compressed spinal cord and posterior instrument stabilization in all cases. The tumor surrounding the spinal cord was removed to the extent that was possible until the decompression of spinal cord was confirmed. The stabilization levels were basically two levels above and two levels below the decompressed spine cord level, but the range of stabilization was extended depending on the situation.

### Statistical analysis

The Chi-squared test and Wilcoxon’s signed-rank test were used for group comparisons. The overall survival curves were calculated by the Kaplan–Meier method and compared by Wilcoxon’s test. *P* values < 0.05 were considered to be significant for all statistical tests.

## Results

### Patient characteristics

The mean age was 61 years (29–81) in the radiation group and 64 years (45–87) in the surgery group. The mean SINS was 10.0 (4–14) in the radiation group and 10.9 (5–18) in the surgery group. The mean Tokuhashi score was 7.0 (3–11) in the radiation group and 8.1 (1–14) in the surgery group. The Frankel grade was A in 1, C in 8, and D in 11 (better than D: 55%) in the radiation group, and C in 16 and D in 18 (better than D: 52%) in the surgery group. The location of spinal metastasis was in the cervical spine in 1 patient and in the thoracic spine in 19 patients in the radiation group and in the cervical spine in 1 patient and in the thoracic spine in 33 in the surgery group. Table 2 summarizes this information. There were no significant differences in the Tokuhashi score, SINS, or Frankel grade between the two groups.

**Table 2:** Characteristics of patients in the radiation and surgery groups

	Radiation group	Surgery group	*P*
Age	61 (29–81)	64 (45–87)	0.67
Sex	M 14 F 6	M 17 F 17	0.17
Tokuhashi score	7.0 (3–11)	8.1 (1–14)	0.11
SINS	10.0 (4–14)	10.9 (5–18)	0.17
Frankel grade	A1 C8 D11 better than D 55%	C16 D18 better than D 52%	1.0
Treatment history	(+)14 (−)6	(+)24 (−)10	0.76
Radiation history	(−)20	(+)6 (−)28	
Level	Cervical 1 thoracic 19	Cervical 1 thoracic 33	1.0

The primary tumor histologies were breast (*n* = 2), lung (*n* = 3), prostate (*n* = 4), liver (*n* = 2), kidney (*n* = 3), gastrointestinal (*n* = 2), other genitourinary (*n* = 1), sarcoma (*n* = 2), and others (*n* = 1) in the radiation group. In the surgery group, the primary tumor histologies were breast (*n* = 4), lung (*n* = 4), prostate (*n* = 4), thyroid (*n* = 3), liver (*n* = 1), kidney (*n* = 1), pancreas (*n* = 2), gastrointestinal (*n* = 5), sarcoma (*n* = 5), unknown (*n* = 2), and others (*n* = 3).

### Neurological outcomes and the survival

**Fig. 1:**
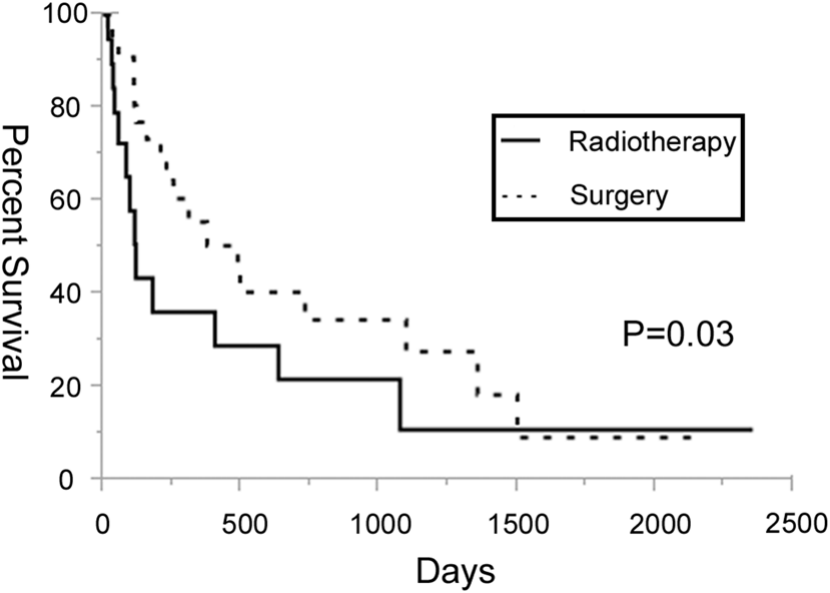
Kaplan–Meier estimates of the survival after treatment

Seven patients initially in the radiation group underwent surgery because of a substantial decline in their motor strength during radiotherapy. One of the remaining 13 patients (8%) in the radiation group and 30 of the 34 patients (88%) in the surgery group showed improvement in their neurological symptoms (*P* < 0.01). One patient (8%) in the radiation group and 21 patients (62%) in the surgery group were ambulatory after treatment (*P* < 0.01). Four of the seven patients converted to surgery in the radiation group showed improvement in their neurological symptoms. One of 8 (13%) patients in the radiation group and 6 of 16 patients (38%) in the surgery group who were initially not ambulatory regained the ability to walk after treatment (*P* = 0.352) (Table 3). The overall survival curves are shown in Fig. 1. The 50% survival rate was 113 days in the radiation group and 365 days in the surgery group (*P* = 0.03).

**Table 3:** The neurological outcomes in the radiation and surgery groups

	No.	Improve	Ambulate (discharge)
Radiation group			
Not ambulate	8	1/8	1/8
Ambulate	5	0/5	0/5
Total	13	1/13 (8%)	1/13 (8%)
Surgery group			
Not ambulate	16	15/16	6/16
Ambulate	18	15/18	15/18
Total	34	30/34 (88%)	21/34 (62%)

### Complications

In the radiation group, death from rupture of liver metastasis occurred in one patient. In the surgery group, there were complications in 9 of 34 (26%) patients. The details were as follows: postoperative paraplegia in one, hematoma in two, wound infection in three, postoperative pneumonia in two, and gastric perforation in one patient. The patient with postoperative paraplegia developed Frankel A status and did not recover. Reoperation was performed in 6 patients (18%) for postoperative paraplegia, hematoma, and wound infection. The mean blood loss was 1072 g (110–4430), and the mean operating time was 259 min (152–527).

### Case presentation 1 (recovered with radiotherapy alone)

**Fig. 2:**
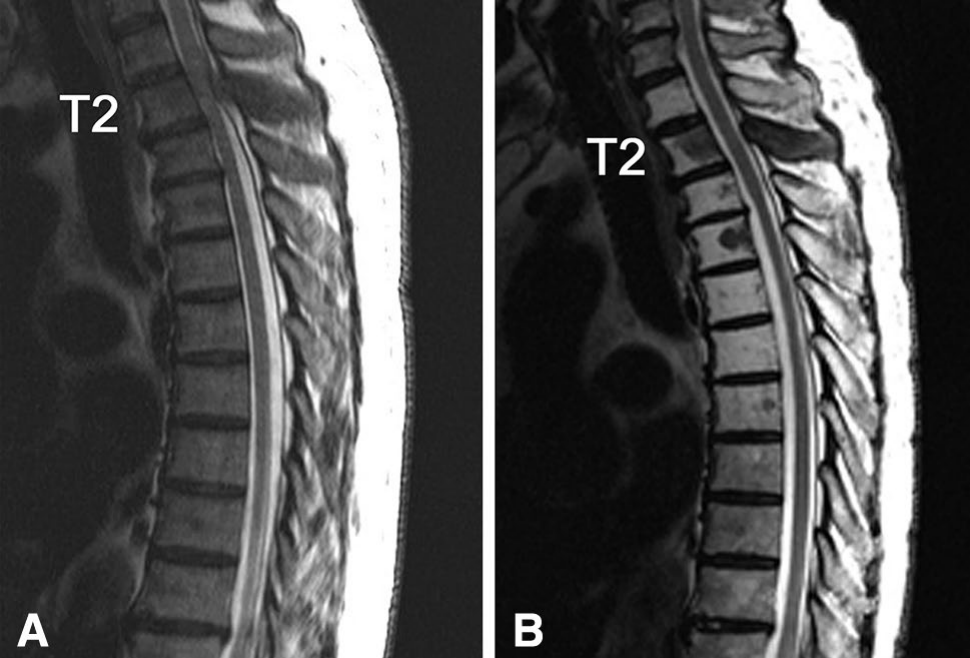
The images of thoracic column in the patients with MSCC of prostate cancer. The tumor in the second thoracic vertebra was improved by radiotherapy.** a** T2-weighted sagittal MRI images before the radiotherapy.** b** T2-weighted sagittal MRI images 1 year after the radiotherapy

A 70-year-old male with leg paralysis was introduced to our institution. He could not flex his hips and knees and was diagnosed with Frankel C1 status. MRI revealed a tumor in the second thoracic vertebra and spinal cord compression. His Tokuhashi score was 11/15, and the SINS was 4/18. He was diagnosed with prostate cancer by a prostate biopsy and treated by radiotherapy and hormone therapy. The tumor size was reduced by the treatment, and he became able to walk with a cane. At 6 years after treatment, he is still alive (Fig. 2).

### Case presentation 2 (surgery performed during radiotherapy)

**Fig. 3:**
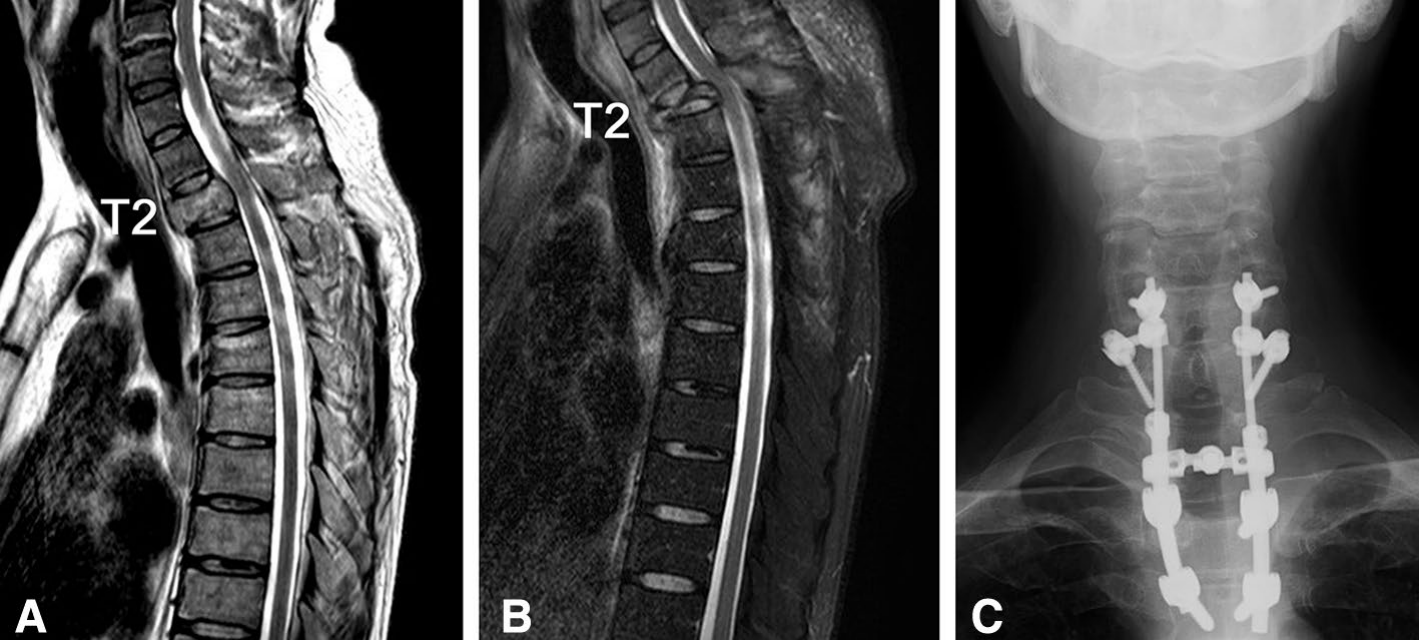
The images of the thoracic column in the patients with MSCC of esophageal cancer. The second thoracic vertebra collapsed, and surgery was performed.** a** T2-weighted sagittal MRI images before the radiotherapy.** b** T2-weighted fat suppression sagittal MRI images when the thoracic vertebra collapsed.** c** Anteroposterior plain radiograph after surgery

A 45-year-old male with back pain was introduced to our institution. He had a little numbness in his foot but was able to walk independently. He was diagnosed with Frankel D3 status. MRI revealed a tumor in the second thoracic vertebra and spinal cord compression. His Tokuhashi score was 8/15, and the SINS was 14/18. He was diagnosed with esophageal cancer by gastroscopy and treated by radiotherapy alone. The second metastatic thoracic vertebra collapsed during the radiotherapy, and he lost the ability to walk. Emergency surgery was performed, and he became able to walk with a cane. He died 1 year after the surgery (Fig. 3).

## Discussion

Radiotherapy has been established as safe and effective for the treatment of MSCC [12, 13]. Radiotherapy is effective for relieving pain due to bone metastasis, with a reported overall response rate for pain of about 60% and a spinal cord compression rate of about 5% after radiotherapy [13]. However, the optimum treatment for patients presenting with neurological symptoms is controversial. Patchell et al. reported that surgery followed by radiotherapy was superior to radiotherapy alone in restoring the ability to walk, increasing the duration of ambulatory status, and improving the survival in their randomized control trial [2]. In contrast, Rades et al. claimed that the outcome of ambulation status did not significantly differ between patients receiving radiotherapy alone and those receiving surgery plus radiotherapy in their matched pair analysis [5]. Many studies have explored the clinical outcomes of MSCC, and in general, the surgical excision of the tumor and instrumented stabilization seems to lead to favorable neurological outcomes more often than radiotherapy alone [2, 3, 14].

Patchell’s randomized control trial has been criticized due to the low efficiency of radiotherapy [2, 5]. Of note, however, their trial only involved patients with MSCC in the cervethoratic region, whereas other studies did not match the region when comparing the neurological outcomes between radiotherapy and surgery groups. Such differences in the inclusion criteria of patients tend to produce differences in the results. Our research concerns patients with MSCC in the cervethoratic region, just like Patchell’s report, and similarly shows the inferior effectiveness of radiotherapy compared with surgery. The cervical region was involved in only two cases in the present study. This is not because cervical metastases are rare but because most of the patients with involvement of the cervical region had radiculopathy or neck pain or spinal instability. These symptoms were exclusion criteria in our study, and patients presenting with cervical radiculopathy with MSCC improved by radiotherapy were not included in our study. These patient selection criteria were one of the reasons for the relatively low effectiveness of radiotherapy noted in our study. These findings suggest that the compression level of the spinal cord (cervethoratic or lumbar) and the symptoms (myelopathy or radiculopathy) should be clarified when comparing the outcomes of treatment.

Seven of 20 cases selected to receive radiotherapy in our study had no benefit from the therapy and were converted to surgical treatment. Surgical decompression is immediate, but radiotherapy requires time to have an effect, and the efficacy depends on the tumor type. If the duration of spinal cord compression is short, the neurologic symptoms are reversible, but if the duration is long, the lengthy compression induces infarction of the spinal cord with secondary vascular injury, and neurologic recovery becomes difficult [2]. When radiotherapy is ineffective, the paralysis proceeds, and the complication rate of surgery becomes higher than with surgery followed by radiotherapy [2]. Our results suggest the need for acute direct decompression of the spinal cord in patients presenting with myelopathy to ensure the resolution of neurological symptoms.

The neurological recovery rate was only 8% by radiotherapy alone in our study. The only patient who recovered with radiotherapy alone had prostate cancer and no treatment history. The metastatic spinal vertebra was osteosclerotic and presented with little instability. As mentioned in previous reports, hormonal therapy with external radiation may be a feasible option in untreated prostate cancer patients [15]. Such patients with tumors highly sensitive to medical therapy may be able to be treated by radiotherapy alone if the spinal column does not show instability [16, 17]. However, there have been no reports comparing the outcomes between surgery and radiotherapy in certain radiosensitive tumors. Further studies are needed to determine the optimum treatment for different tumor types.

The complication rate was very high in the surgery group in our study, just as mentioned in previous reports [4]. This is due to the poor general condition of such patients and the invasiveness of surgery. Patients undergoing surgery are generally not in a good general condition, and the operative blood loss is excessive due to the hypervascularity of tumors. Reducing the invasiveness is important for reducing the rate of complications. All of the patients who saw no improvement in their neurological symptoms in the surgery group had operative complications in our study. A minimally invasive surgery procedure, such as percutaneous fixation, may reduce the operative invasiveness and reduce the rate of complications [18].

Our study is a retrospective study, so there are large selection biases. The decisions regarding treatment in this study were based on individual experience, thus a selection bias cannot be excluded. To show whether or not any bias was associated with our study, we compared the Tokuhashi score, SINS and Frankel grade. There were no significant differences between the prognosis, spine column instability, or progress of paralysis between the two groups. However, the overall survival was significantly different between the radiation and surgery groups. This difference of survival probably does not show the surgery improved the survival, but shows the difference of general condition between the groups. Some patients considered unable to tolerate surgical invasion may have been selected to receive radiotherapy alone. The other limitation is the short-term observation period for evaluating neurological recovery. We could not evaluate the recurrence of neurological symptoms. Our surgeries were all palliative in nature; thus, the tumor could grow and paralysis could proceed with the progression of primary cancer. However, we considered surgery to be important for MSCC patients because the performance status is a key factor for the decision-making regarding chemotherapy. Patients with metastasis are generally treated by chemotherapy; however, patients who could not walk tended to be selected for palliative treatment. Once a patient’s performance status is improved, the patient receives chemotherapy and tumor progression can be suppressed. Such factors may have also contributed to the difference between the groups with regard to survival.

Patients with MSCC present with several pathological conditions, and we only described the treatment of highly select patients in the present study. The surgical decision was comprehensively determined by not only considering the patient’s potential neurological recovery but also their general condition and prognosis. However, to recover the neurological function, surgery may be necessary in MSCC patients presenting with myelopathy.

## Conclusions

In this retrospective series, radiotherapy alone was less effective than surgery in MSCC patients presenting with myelopathy. Surgery may be necessary to recover the neurological function, but the high rate of complications must be considered. In addition, the symptoms and compression level of the spinal cord should be mentioned when comparing the neurological outcomes between radiotherapy and surgery, as the ideal treatment differs by situation.
